# Artificial Neural Network Application for Current Sensors Fault Detection in the Vector Controlled Induction Motor Drive

**DOI:** 10.3390/s19030571

**Published:** 2019-01-29

**Authors:** Mateusz Dybkowski, Kamil Klimkowski

**Affiliations:** 1 Department of Electrical Machines, Drives and Measurements, Wroclaw University of Science and Technology, ul. Wybrzeze Wyspianskiego 27, 50-370 Wroclaw, Poland; 2 Independent Researcher, 50-370 Wroclaw, Poland; kamil.klimkowski89@gmail.com

**Keywords:** induction motor, Direct Field Oriented Control (DFOC), Fault Tolerant Control (FTC), neural network, current sensor, fault detector

## Abstract

This paper describes a Fault Tolerant Control structure for the Induction Motor (IM) drive. We analyzed the influence of current sensor faults on the properties of the vector-controlled IM drive system. As a control algorithm, the Direct Field Oriented Control structure was chosen. For the proper operation of this system and for other vector algorithms, information about the stator currents components is required. It is important to monitor and detect these sensor faults, especially in drives with an increased safety level. We discuss the possibility of the neural network application in detecting stator current sensor faults in the vector control algorithm. Simulation and experimental results for various drive conditions are presented.

## 1. Introduction

The efficiency and performance levels of electrical machines gradually deteriorate as a result of their wearing and aging processes. Therefore, the reliability of entire technological processes decreases, which simultaneously increases the risk of basic system components failure [1]. The most common examples of well-known abnormalities are actuator lock, partial or full loss of sensor signal, short circuits, and system component sudden disconnection [2]. These may cause intermittent operation of the controllers or significant measurement errors. Consequently, this leads to reduced system performance and even to total system failure. In order to prevent damage, or to enable early detection, various diagnostic techniques are used and tested [1], including adequate protection of key components, and redundancy and technical diagnostics [3,4,5,6,7,8].

To ensure the safe and efficient operation of any industrial process regardless of its complexity, it is necessary to continuously monitor its operation by means of special measuring devices. The growing demand for increasingly reliable high performance installations, due to increased security regulations and a more competitive market [1,4,5], requires the implementation of effective diagnostic systems, such as Instrument Fault Detection and Identification (IFDI) [3,6], Fault Detection and Diagnosis (FDD) [3], and Fault Tolerant Control Systems (FTCS) [3,6,8,9].

Increased system reliability can be achieved through the implementation of additional diagnostics that perform three essential functions [1,6]: detection, identification, and isolation of a fault. In automatic control systems, the failures of actuators, control systems, and measuring systems are considered. In practical applications, diagnostic systems monitor and identify faults by applying hardware redundancy or software redundancy [1,5,7,8]. 

Hardware redundancy uses several identical executive, control, or measurement elements with the same input signal to compare their responses [10].

The occurrence of the failure is determined based on the analysis of the difference between the received output signals. Fault isolation is performed by turning off the faulty element and using one of the correct components. Because of their simplicity and reliability, this remains the most frequently used solution [1,5,6,7,8,9,10]. Their major drawback is the application of additional hardware, which increases operational costs. In practical applications, due to its high cost and limited work space, only key components can be duplicated. 

The development of control theory led to the development of diagnostic techniques based on analytical redundancy [1,5,10,11,12,13]. Diagnostic methods based on the concept of analytical redundancy do not require additional components, but rely on mutual relationships between measured and available signals [1,5,10]. Based on the input and output signals, it is possible to detect and identify failures in real time during system operation. 

Compared with hardware redundancy techniques, analytical methods are more efficient and cheaper, but simultaneously more difficult in practical implementation. The main problem is the processing of the measured signals, their quality, and the susceptibility to external signals [11]. In addition, complex control systems require more complex diagnostic methods. Based on this information, monitoring techniques can be divided into three categories [1,3,10]: mathematical model-based methods, signal analysis-based techniques, and heuristic methods.

The mathematical models of the analyzed objects are used in the diagnostic and monitoring approach. This is one of the most important tasks in those systems.

The first solution, based on the mathematical models (MM), was proposed by Beard [14] in 1971. It was an alternative system for the well-known hardware redundancy methods [1,15,16]. Methods based on MM require information about the physical model of the observed system [3,10]. This developed mathematical model is used for developing fault diagnostic and monitoring algorithms [3,10,17,18,19,20]. The mathematical model of the developed system should be equal to the real model. The mathematical model should reflect the actual system as accurately as possible. Diagnostic methods based on mathematical models provide ample development opportunities as they are based mainly on various techniques of state variables estimation, which are currently popular. In addition, the rapid progress in the computer science and electronics fields, especially related to digital signal processors and field programmable gate array (FPGA) systems with high computing power, has enabled the application of a combination of algorithms based on mathematical models and other diagnostic methods, e.g., fuzzy logic, neural networks, or statistical techniques. This has resulted in the increased reliability of these systems while reducing their sensitivity to noise, ensuring high quality performance and security of complex industrial applications. The issue of monitoring and detection of failures based on mathematical models is a well-developed subject of research [20].

Another group of diagnostic methods used in drives applications are algorithms based on signal analysis (SA) [3,20]. They are based solely on advanced mathematical tools and data processing techniques for extracting essential features that are fundamental for a diagnostic evaluation of object condition. The occurrence of any failure in the monitored system is reflected in the measured signals, which facilitates diagnostic decisions. Relevant information from measurements is extracted by suitable processing tools, whose choice is highly dependent on the type of the monitored object. Signals can be analyzed in a continuous or discrete time domain [20,21,22,23]. Diagnosis based on signal analysis methods requires knowledge about the possible faults symptoms and the interpretation of current data obtained from measurements and their analysis [21]. 

Basic SA monitoring methods are based on the classic inference rules, where expert knowledge on the diagnosed object and its ability to perceive the characteristic features of the variables describing various states of system operation are crucial [21]. Modifications of these methods with additional units of analysis and assessment of fault symptoms based on the theory of artificial intelligence (neural networks—NN, fuzzy logic—FL, and neural-fuzzy networks—NFN) are becoming increasingly popular [24,25,26,27,28,29,30,31,32,33]. These systems produce better evaluation and accurate diagnosis possibilities of the inference system based on the characteristic properties of the measured signals [19].

**Figure 1:**
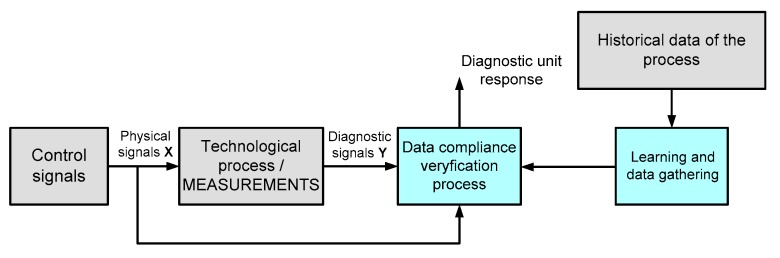
Data analysis based diagnostic system—general diagram.

Diagnostic techniques based on the heuristic approach have been mainly used in complex applications that have rich databases containing relevant information about the operating conditions of the system in the past. Based on this knowledge, various methods for the extraction of intermediate dependencies between individual variables of the monitored process state and failure symptoms may be applied [29,30,31]. Fault diagnosis is conducted by checking the conformity of the collected (historical) and currently measured data with the entire system. Heuristic knowledge includes observation, inspection, and collecting information in various forms: tones, vibrations, colors, temperatures, and signal amplitudes. The key source of this knowledge is the history of a given process, and above all: previous failures, maintenance, and repair operations experiences as well as statistical data collected from similar objects [3]. Notably, diagnostic algorithms based on heuristic knowledge use huge amounts of process data in real time and process the data online. For this reason, these methods are defined in the literature as techniques based on data analysis. A schematic diagram of these diagnostic methods is presented in Figure 1.

In the methods based on data analysis, the fuzzy logic theory may be implemented in diagnostic systems due to its high applicability, especially in the inference process and in decision-making units [7,20]. These techniques are usually used for the advanced analysis of measured signals carrying essential information about the state of the monitored object [26]. In addition, hybrid systems [31,32] based on various methods of data analysis, e.g., neural networks and fuzzy logic, are increasingly being designed and analyzed in order to obtain a more effective and faster diagnosis of failure [33].

One of the most complex diagnostic methods is the technique based on artificial neural networks (ANN) for the faults symptoms extraction of a specific sensor [12,34]. Designed ANN detectors are trained on the basis of monitored transients of chosen measured signals in a wide range of the motor speeds. At appropriate time points, the failure of the tested sensor can be simulated. Based on the received teaching vector, the neural network is able to identify failure occurrence in real time. In this method, neither the mathematical model of the system nor the calculation of threshold limits for diagnostic signals are needed.

One of the disadvantages of this approach is the requirement properly choosing the teaching vector that should contain as much information as possible about the monitored object state variable in both normal and emergency operation. In the neural network design process, its structural topology, number of hidden layers, neurons in each layer, and the choice of the learning algorithm also play a crucial role. Designing a properly functioning fault detector based on a neural network is not an easy task and requires considerable experience and knowledge about the diagnosed object and the theory related to artificial intelligence [35,36,37,38].

Thus, the main goal of this paper was to demonstrate a stator current transducers fault detection algorithms for the vector control (DRFOC) of an induction motor drive system based on an active detection system [20]. The proposed system guarantees stable operation of the drive during faulted conditions. This system is based on an artificial neural network trained with signals obtained from the internal control structure. The diagnostic method was analyzed and tested in different drive operation conditions. The simulations and experimental results of the proposed Fault Tolerant Control are presented.

## 2. Current Sensor Faults Analysis 

**Figure 2:**
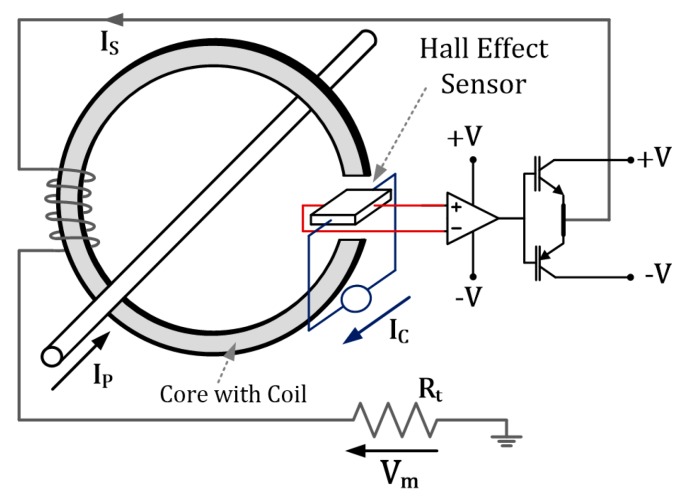
Current sensor scheme based on the Hall effect.

A stator current sensor is necessary for the proper operation of electrical drives controlled using vector control algorithms [3,4]. Stator current signals are used in the internal control loop for state variable estimation [2,39] and in the internal control loop. One of the most popular transducers, used in industry and research, is the closed-loop hall-effect current sensor presented in Figure 2.

Basic fault types of the stator current sensor are presented in Table 1.

**Figure 3:**
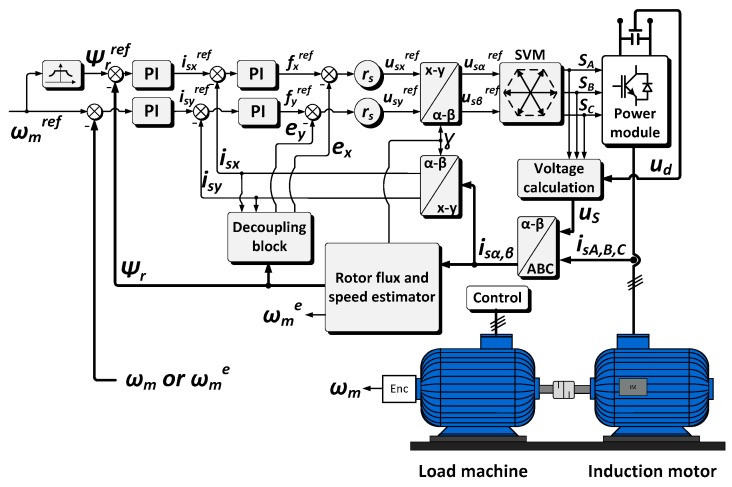
General scheme of the Direct Rotor Field Oriented Control (DRFOC).

In the next part of the paper, the influence of broken stator current sensor (in phase A) on the performance of the motor drive is shown and described. The selected fault cases were examined simulation software upon the well-known Direct Field Oriented Control (DFOC) structure for induction motor (shown in Figure 3). 

An incremental encoder was applied to measure speed (5000 imp/rev). The direct current (DC) bus voltage and the IGBT (insulated gate bipolar transistor) states were used to calculate the stator voltage components. Stator currents in the healthy condition of the drive were measured by the two closed-loop halleffect current sensors in phases A and B (Figure 2). The third transducer can be used only in the sensor failure scenario. 

Information about the rotor flux is necessary for the proper operation of the drive. This value can be obtained by a simulator based on the current model of a rotor flux:

(1) $$\frac{d}{dt}\Psi_{r}^{i}=\left[\frac{r_{r}}{x_{r}}(x_{m}i_{s}-\Psi_{r}^{i})+j\omega_{m}^{}\Psi_{r}^{i}\right]\frac{1}{T_{N}},$$

For the rotor speed estimation, the Model Reference Adaptive System (MRAS^CC^) estimator can be used [36,40]. The current estimator used in MRAS^CC^ can be obtained by:

(2) $$\frac{d}{dt}i_{s}^{e}=-\frac{r_{r}x_{m}^{2}+x_{r}^{2}r_{s}}{\sigma T_{N}x_{s}x_{r}^{2}}i_{s}^{e}+\frac{1}{\sigma T_{N}x_{s}}u_{s}+\frac{x_{m}r_{r}}{\sigma T_{N}x_{s}x_{r}^{2}}\Psi_{r}^{i}-j\omega_{m}^{e}\frac{x_{m}}{\sigma T_{N}x_{s}x_{r}^{}}\Psi_{r}^{i},$$

where $\omega_{m}^{e}$ is the estimated rotor angular speed; $r_{s}$, $r_{r}$, $x_{s}$, $x_{r}$, and $x_{m}$ are the stator and rotor resistances, stator and rotor leakage reactances, and mutual reactance; $u_{s}^{}$, $i_{s}^{e}$, and $\Psi_{r}^{i}$ are the stator voltage, estimated stator current, and rotor flux vectors, respectively; $\sigma =1-x_{m}^{2}/x_{s}x_{r}$; and $T_{N}=1/2\pi f_{sN}$.

The current model in Equation (2) and rotor flux model in Equation (1) are used in the adaptation mechanism for speed reconstruction [3]:

(3) $$\omega_{m}^{e}=K_{P}\left(e_{i_{s\alpha }}\Psi_{r\beta }^{i}-e_{i_{s\beta }}\Psi_{r\alpha }^{i}\right)+K_{I}\int \left(e_{i_{s\alpha }}\Psi_{r\beta }^{i}-e_{i_{s\beta }}\Psi_{r\alpha }^{i}\right)dt,$$

where $e_{i_{s\alpha ,\beta }}=i_{s\alpha ,\beta }-i_{s\alpha ,\beta }^{e}$ is the error between the estimated and measured stator current.

**Figure 4:**
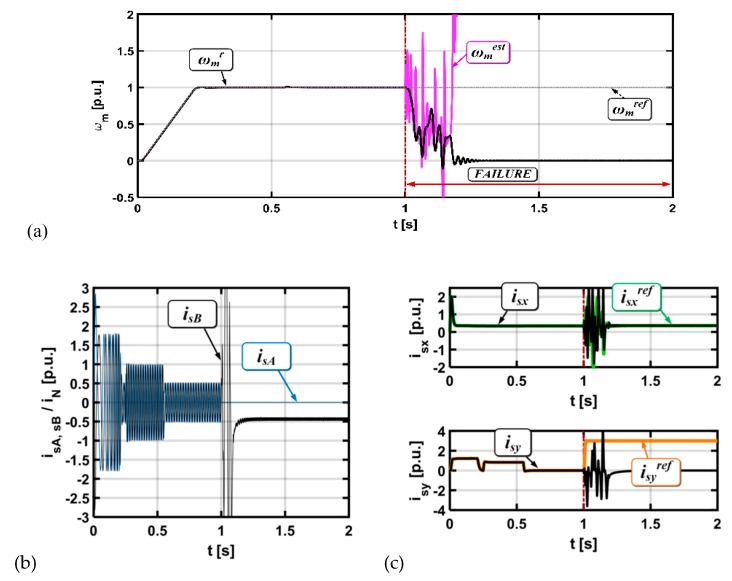
Results for the drive system with a broken current sensor in phase A (lack of signal), (rotor speed (**a**), stator current (**b**), stator current components x,y (**c**)), *i_A_^’^ = 0.*

The mathematical model and detailed analysis (including stability analysis [36,41]) of the MRAS^CC^ estimator was described in detail [36]. Figure 4 presents the simulation analysis for the vector controlled drive system, with a broken current sensor in phase A (lack of signal). The current sensor was destroyed (in time t = 0.5 s). The effects of this fault are visible on all the analyzed transients.

**Figure 5:**
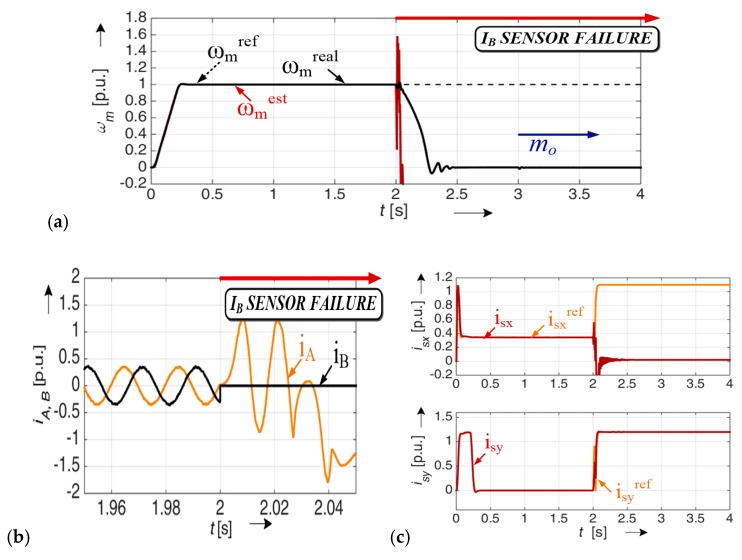
Results for the drive system with a broken current sensor in phase B (lack of signal), (rotor speed (**a**), stator current (**b**), stator current components x,y (**c**))*, i_B_^’^ =* 0.

Figure 5 presents a similar test. We assumed that in this test the current sensor in phase B is faulted.

The current sensor was broken for *t =* 1 s (Figure 4) and for *t =* 2 s (Figure 5). 

The control structure cannot compensate for the behavior of the drive after faults. The fault symptoms are also visible in the measured and estimated speed values and in the stator current components transients. 

**Figure 6:**
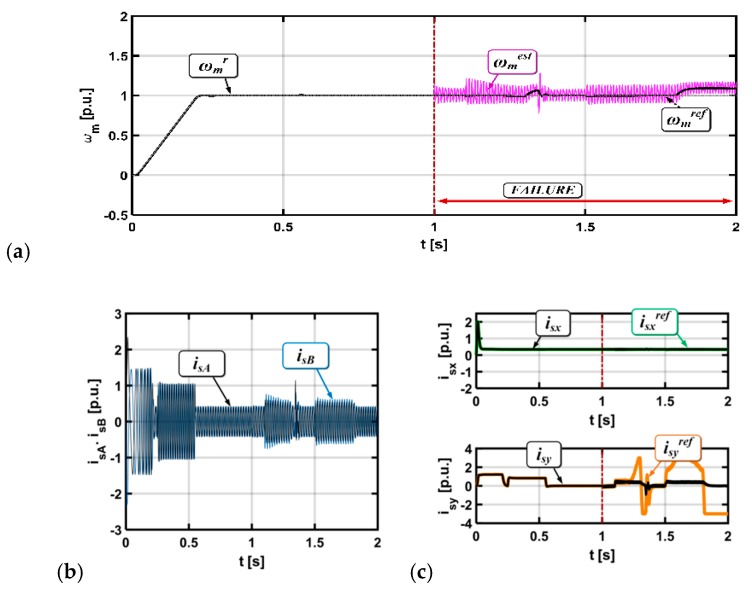
Results for the drive system with a broken current sensor in phase A (gain error), (rotor speed (**a**), stator current (**b**), stator current components x,y (**c**)).

**Figure 7:**
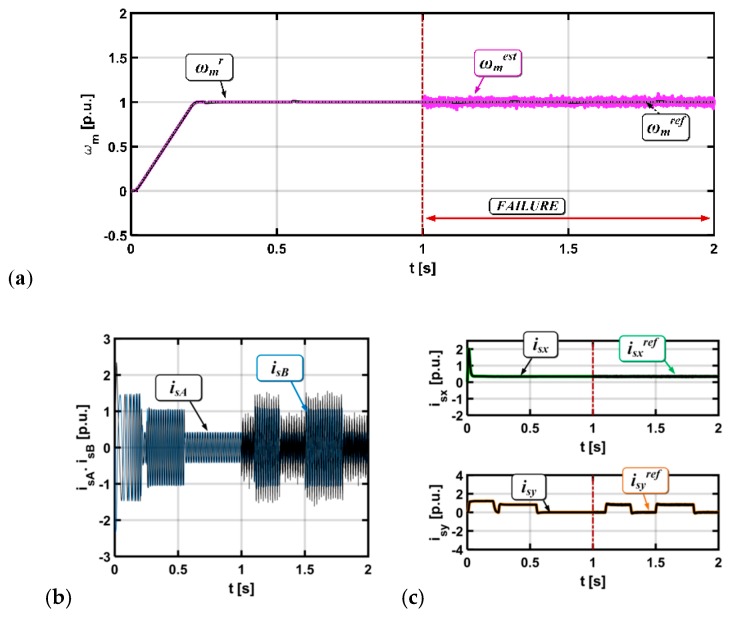
Results for the drive system with a broken current sensor in phase A (white noise), (rotor speed (**a**), stator current (**b**), stator current components x,y (**c**)).

Figure 6 and Figure 7 present state variable transients for the induction motor drive operation with a gain error and with white noise, respectively. 

For the stator current errors, oscillations in the measured and estimated speed can be observed. For both types of faults, the electrical drive is stable and can work properly with a broken sensor.

The lack of signal from the stator current sensors is the most dangerous fault for the drive. It is extremely harmful for the stability and safety of operation. It is necessary to implement other diagnostic systems to prevent these situations.

## 3. Fault Tolerant Control (FTC) Analysis

**Figure 8:**
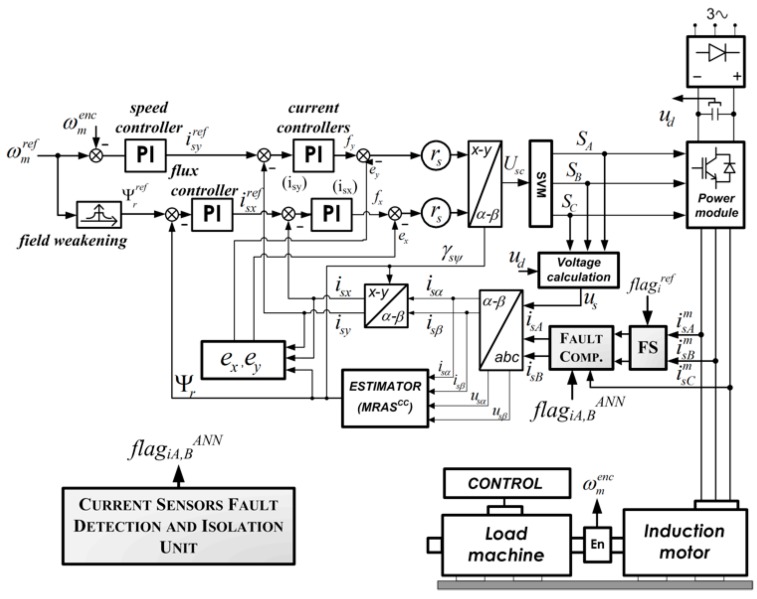
Scheme of the Direct Field Oriented Control (DFOC) structure with current sensor fault detection algorithm.

This part of the paper presents a detector based on the neural network (NN) for stator current sensor faults in the DFOC drive. The complete scheme of the FTC structure with an additional detection algorithm and a fault compensation mechanism is presented in Figure 8.

**Figure 9:**
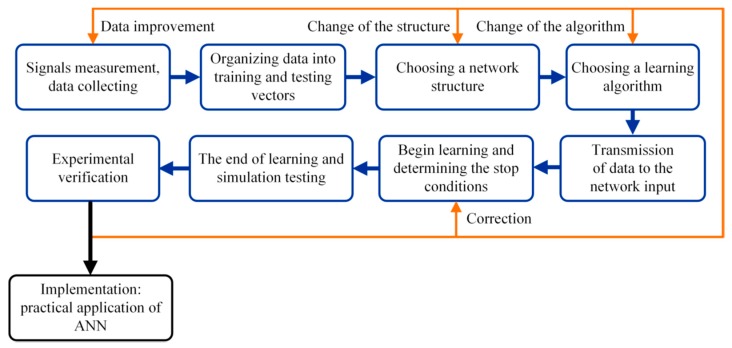
Neural network design cycle [17].

Designing the process of an artificial neural network for diagnostic purposes consists of several basic stages carried out cyclically in a multi-loop structure (Figure 9), in which the following elements can be distinguished:

(1) Defining the types of analyzed faults;
(2) Determining the normal state of system operation (without faults) and emergency state operation of the drive (after failure occurrence);
(3) Designing a neural fault detector: selection of learning signals, network structure, and learning algorithm; simulation testing using computer simulators of neural networks (e.g., MATLAB Neural Network Toolbox); and
(4) Implementation of a neural network on a real object and its experimental testing.

The first, and main stage of the NN design process is the proper selection of learning signals. In the analyzed case of the measurement sensors fault-tolerant system, the state variables, whose failure affects the system directly or indirectly but substantially, were used. Thus, for the DRFOC structure, the following signals were considered: Abs(*i_sy_-i_sy_^ref^*), Abs(**Ψ***_r_^est^-***Ψ***_r_^ref^*), and stator currents *i_sA_*, *i_sB_*.

**Figure 10:**
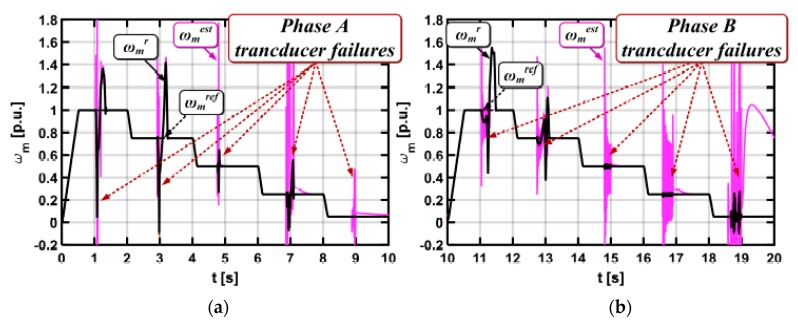
Measured, real, and estimated induction motor speed during the neural network training process (for phase A transducer failures (**a**) and for phase B transducer failures (**b**)).

The next step is to define the cycle of motor drive operation in a wide range of speed changes, during which NN will be taught the emergency states. The cycle of work shown in Figure 10 was used to teach the neural network. 

At the beginning, the system was started at the nominal speed. The speed was reduced by 20% after 2 s of drive operation. For the drive operation, the stator current sensor fault was simulated (the total interruption). Figure 10 depicts the transients of the measured, real, and estimated motor speed during described operation.

The proper selection of a neural network type, an appropriate number of hidden layers, and neurons is one of the most important tasks. 

For diagnostic applications in electric motor drives, multilayer feedforward networks and self-organizing Kohonen’s neural networks [17] are used most often. 

In this paper, a multilayer feedforward network is used, characterized by a lack of feedback connections between individual layers and neurons, layered placed neurons, and a unidirectional information flow. The main task of the neurons in the input layer is the preprocessing of signals, whereas information decision processing occurs in the hidden and output layers. The network’s response is obtained from the output of the neurons in the last layer. Choosing the proper structure for a multilayer neural network is a difficult task because there are no strictly defined criteria and rules for determining the optimal network configuration. Thus, this stage of the NN design process was completed using the experimental method. The research was conducted for networks with two hidden layers and different numbers of neurons in particular layers. The simulation tests included five NN structures based on the constructive principle [2], which aims to start the NN learning process with a small number of neurons and gradually increasing it until the best results are obtained:

(1) (N) - (2N+1) - (2) - (1),
(2) (N) - (2N+1) - (4) - (1),
(3) (N) - (2N+1) - (8) - (1),
(4) (N) - (2N+1) - (10) - (1), and
(5) (N) - 2(2N+1) - (2) - (1),

where N is the number of neurons in the input layer.

The Levenberg-Marquardt (L-M) algorithm [16,33,34] was used for the learning process. This algorithm combines the convergence of the Gauss-Newton algorithm near the minimum and the method of gradient descent for the greater distance from the minimum [42]. The L-M algorithm is one of the most reliable and fastest algorithms used in different applications. The disadvantage of this method is that the memory requirements increase proportionally with the square of the number of weights in the network [37,38,42]. The L-M algorithm uses a compromise learning strategy between the classical linear approach and the gradient method approach in each iteration. Moving the point of seeking the optimum weight is acceptable only if it leads to error reduction [2].

In the next step, the obtained NN detectors were tested for three different drive operation modes with: (1) nominal rotor speed value without the load torque, (2) nominal rotor speed value and load torque value, and (3) low rotor speed value without the load torque.

In each of the above cases, the operation cycle lasts 2 s, and the moment *t =* 1 s the total failure to the current transducer is simulated. To assess the effectiveness of the designed neural networks, two quality indicators: ITSE (integral of square of the error) and ITAE (Integral time absolute error), and detection time Δ*t_det_* were used. ITSE and ITAE are described by dependencies in Equations (4) and (5), respectively:

(4) $$ITSE=\frac{1}{n}\sum_{i=1}^{n}\int_{t=0}^{t=2}t\cdot e^{2}\left(t\right)\;dt,$$

(5) $$ITAE=\frac{1}{n}\sum_{i=1}^{n}\int_{t=0}^{t=2}t\cdot \left|e\left(t\right)\right|\;dt,$$

$$\Delta t_{\det}=t_{A}-t_{D},$$

where *e (t)* is the error between reference value failure simulator output signal and detector response, *t_A_* is the time a failure was simulated, and *t_D_* is the time a failure was detected.

The proposed fault detection algorithm is based on a neural network that operates in the open loop without any connection to the control algorithm. This methodology is based on the signals from the internal control structure. Thus, the neural network does not affect the internal control signals or structure in normal operating condition. Before and after fault occurrence the electrical drive is stable because it is based on conventional solutions (classical DFOC algorithm with current model to the rotor flux estimation). The only unstable condition applies to the duration of the failure. The stability of the drive depends on the stability of the flux and speed estimator. This analysis was presented in detail in previous studies [12,19,36,43,44,45,46,47,48]. 

**Figure 11:**
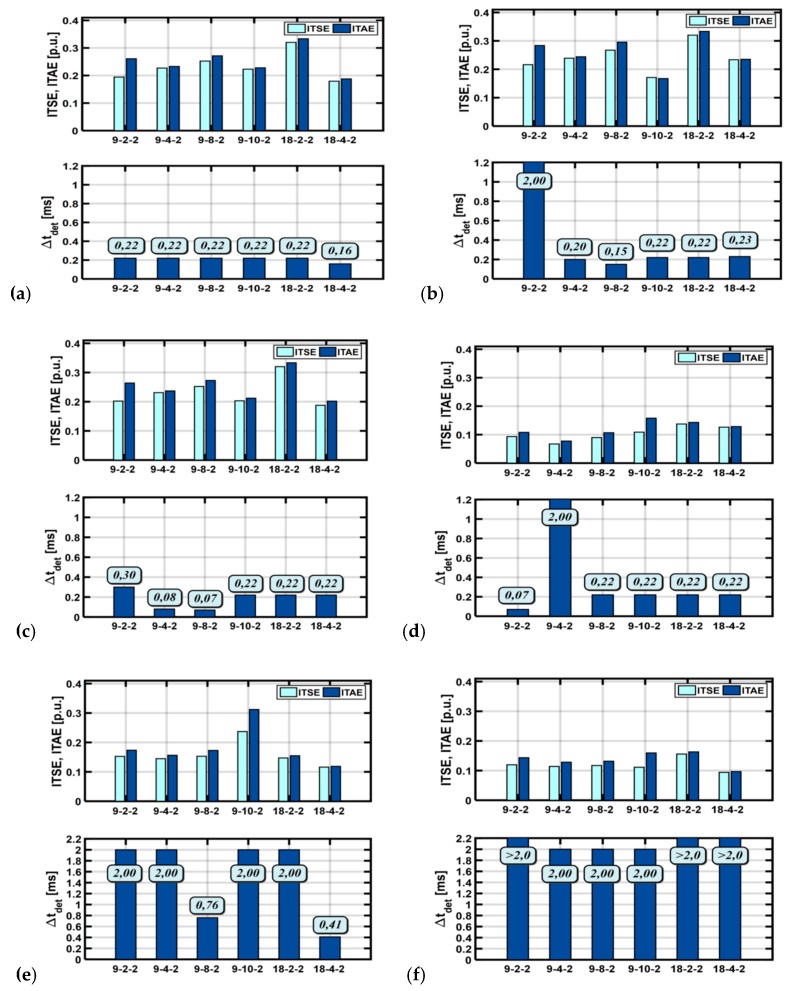
Bar charts with the values of ITSE and ITAE integral quality indicators and fault detection times for different NN learning cycles: (**a**,**d**) operation of the drive with a rated speed value, (**b**,**e**) rated speed and load torque values, (**c**, **f**) low speed value failure detection of the current transducer phase A (**a**, **b**, **c**) and phase B (**d**, **e**, **f**), simulation results.

Figure 11 presents the values of these two quality indicators and the detection time for each of the analyzed neural networks. The obtained results indicate that there is at least one network structure that provides a short detection time (Δ*tdet* = 0.22 ms) and results in low quality indicator values for all drive operation modes. For both the rated speed and its low values, very good results were obtained for each analyzed algorithm. The best result was obtained for the neural network in the configuration [4-9-8-2].

**Figure 12:**
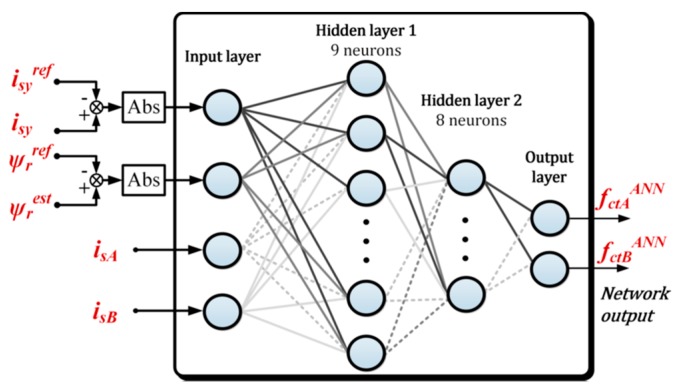
Schematic diagram of designed NN-based detector.

Figure 12 provides a diagram of the proposed neural network current fault detector for the DRFOC vector control structure. On the input layer of the neural network detector, the signals from the internal control loop (from DFOC) are used. The signal (binary) related to the fault is on the NN detector output. 

**Figure 13:**
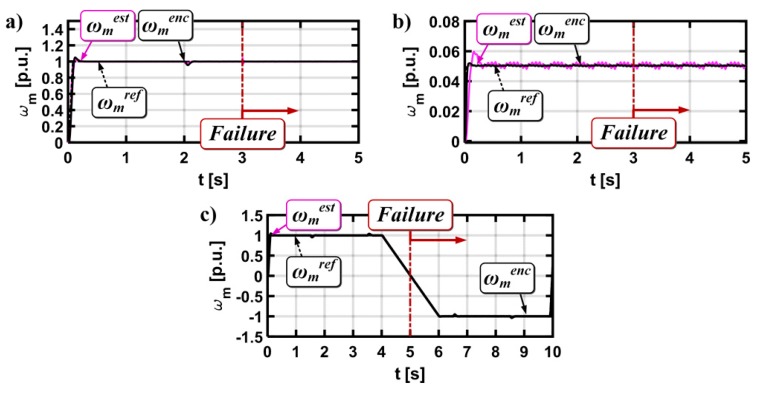
Measured, real, and estimated rotor speed during total failure of the phase A current transducer: (**a**) *ω_m_ = ω_mN_, m_o_ = m_oN_*, (**b**) *ω_m_ = *0.05*ω_mN_*, and (**c**) *ωm = ±ω_mN_, m_o_ = m_oN_* reverse operation—experimental results.

The next step in designing the NN-based detector is its experimental verification. In these tests, the motor was started from zero to reference speed. The stator current sensor was faulted at the moment of time *t =* 3 s. Three fault types were analyzed: total failure of the measuring transducer (Figure 13a), gain error of the current transducer (Figure 13b), and sensors output signal with white noise (Figure 13c).

**Figure 14:**
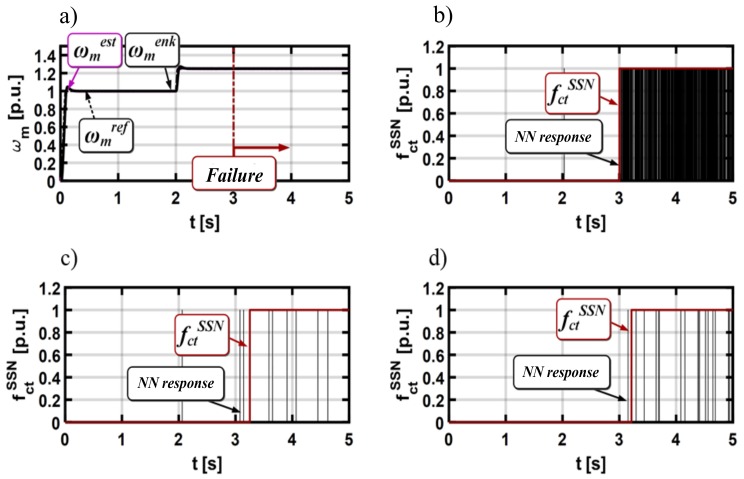
Experimental results of (**a**) measured, real, and estimated rotor speed during: (**b**) total failure of the phase B current transducer, (**c**) white noise, and (**d**) gain change, *ω_m_* = 1.25*ω_mN_*

The drive was also tested for the field weakening region (Figure 14).

**Figure 15:**
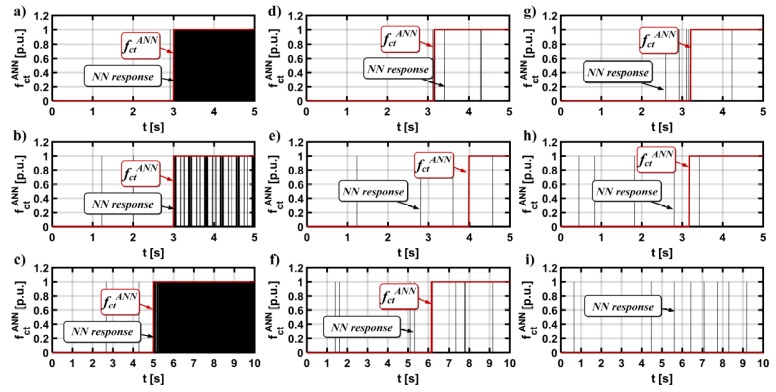
Experimental results of the transients of the output signals from a neural detector during failures of the phase A current transducer: *ω_m_ = ω_mN_*, *m_o_ = m_oN_* (**a**,**d**,**g**), *ω_m_ = *0.05*ω_mN_* (**b**,**e**,**h**), *ω_m_ = ω_mN_*, *m_o_ = m_oN_* reverse operation (**c**,**f**,**i**)—total failure of the sensor (**a**,**b**,**c**), white noise (**b**,**c**,**d**) and gain change (**g**,**h**,**i**).

The experimental tests indicate that, in most cases, the proposed neural network was able to detect the damage of the stator current transducers in phases A and B used in the field-oriented control structure of an induction motor (Figure 15). The most significant failure, which is the lack of signal from the sensor, was quickly diagnosed for each analyzed operating state (Table 2 and Table 3), which allowed for the safe reconfiguration of the measurement system and continuation of operation.

Less significant current sensors failures (such as noise or gain change) had much slower detection times. In some cases, they were not detected at all (symbol ND in Table 2 and Table 3).

This is mainly due to their negligible influence on the state of the engine monitored and provided to the input of the neural network. The exact detection times are shown in Table 2 for the damage results of the A phase current transducer, and in Table 3 for the B phase transducer.

## 4. Conclusions

In this paper, we presented and described the novel neural network based detector. Except for the current sensors fault detection methods used in phase A and phase B, the signals from the internal control structure were used (stator current components, rotor flux vector, stator current in measured phases A and B). The proposed algorithm was trained using the Levenberg-Marquardt (L-M) algorithm. The best results were obtained for the L-M learning algorithm, which is a time consuming method, especially for non-single output neural networks. Thus, a proper methodology for choosing the best neural-network-based detector is needed, which can facilitate and hasten the process were practical implementation. The simulation results for each of the presented NN detectors are compared using dedicated indicators (ITSE, ITAE, and detection time). From those tests, it was possible to choose the best NN detector for a given motor.

The network was only trained for the totally broken current sensor in phases A and B. Different topologies of the neural network were checked and compared. This solution may be used for different control algorithms (for example, Direct Torque Control (DTC-SVM)) after amending the learning signals.

We showed that detection times depend on the topology of the neural network. Not all kinds of current sensor faults can be detected in the vector controlled induction motor drive system.

The most dangerous fault was always detected for different drive operation values over a wide range of reference speeds and load torques.

The proposed solution is based on one neural network detector with two output diagnostic signals. Previous studies focused mainly on separate detectors for each of current transducers. 

## Figures and Tables

**Table 1:** Basic faults types of the current sensor, where *i_s_^m^* is measured current, *i_a_* is the real current, *n(t)* is white noise, *γ* is the constant value from the range <−1,1>, *i_sat_* is the limited current, *ω_offset_* = 10 Hz phase shift, and A is current amplitude.

Type of the Fault	Current Value
Variable gain	$i_{s}^{m}=\left(1-\gamma \right)i_{a}\;$
Phase shift	$i_{s}^{m}=i_{a}+i_{offset}^{}$
Signal limit	$i_{s}^{m}=i_{sat}$
Noise	$i_{s}^{m}=i_{a}+n\left(t\right)$
Lack of signal	$i_{s}^{m}=0$
Intermittent signal	$i_{s}^{m}=\left[0,1\right]$

**Table 2:** Experimental results of detection times Δ*t* (ms) of the current sensor fault in phase A for a NN fault detector (Direct Field Oriented Control structure).

	Type of Operation—Experimental Results (Phase A)			
Fault type	*ω_m_* = *ω_mN_* *m_o_* = *m_oN_*	*ω_m_* = 0.05*ω_mN_*	*ω_m_* = 1.25*ω_mN_*	*ω_m_* = *ω_mN_*, *m_o_* = *m_oN_* (reverse)
Total failure	0.8 ms	0.6 ms	0.4 ms	0.6 ms
White noise	210.6 ms	175.1 ms	1.45 s	1.16 s
Gain change	143.7 ms	987.2 ms	ND	ND

**Table 3:** Detection times Δ*t* (ms) of the current sensor fault in the phase B for the NN fault detector (Direct Field Oriented Control structure) from the experimental tests.

	Type of Operation—Experimental Results (Phase B)			
Fault type	*ω_m_* = *ω_mN_* *m_o_* = *m_oN_*	*ω_m_* = 0.05*ω_mN_*	*ω_m_* = 1.25*ω_mN_*	*ω_m_* = *ω_mN_*, *m_o_* = *m_oN_* (reverse)
Total failure	0.2 ms	8.0 ms	3.0 ms	0.8 ms
White noise	320.1 ms	1.34 s	214.0 ms	2.03 s
Gain change	127.1 ms	ND	252.0 ms	342.0 ms
